# A Case of Spontaneous Rupture of a Coronary Artery

**Published:** 1908-01-18

**Authors:** 


### Illustrative Cases.

### A CASE OF SPONTANEOUS RUPTURE OF A CORONARY ARTERY.

We have already given an example of cardiac infarction with subsequent rupture of the heart. The following is a similar case, but differs in that the heart itself did not rupture, death being due to bleeding from the coronary artery into the pericardium.

The patiênt was a woman of 74, who had no albuminuria, but whose arteries were extensively affected by atheroma. One day she suddenly had an ordinary apoplectic seizure, which left behind it complete but transient right hemiplegia. The lesion was clearly a hæmorrhage in the neighbourhood of the left internal capsule from the giving way of an atheromatous lenticulo-striate artery. The hemiplegia rapidly got less, so that the hæmorrhage was probably a small one. Four days later, when the patient seemed to be going on famously, she suddenly fell dead from no apparent cause. It seemed probable that she had had an increase in her cerebral hæmorrhage, though the end was perhaps rather sudden for this; but at the autopsy the following condition of the heart was found:—

The pericardium was distended with blood clot, which was uniformly moulded over the walls of the heart. The myocardium was fatty and friable, and contained numerous diffuse hæmorrhages into its substance, though there was no breach in the wall of any of the cavities. The aortic and mitral valves were atheromatous, but neither incompetent nor stenosed. The coronary arteries, though pervious, were very atheromatous, and near the bifurcation of the left one there was solution of continuity of its anterior branch. It was clear that the bad coronary arteries had suffered numerous small ruptures, which had caused the interstitial hæmorrhages in the myocardium, and that the sudden syncope and death were due to a larger rupture, from which blood had escaped directly into the pericardium.

It is perhaps surprising that atheromatous coronary arteries do not give way more often in this way, seeing how frequent is the giving way of cerebral arteries similarly diseased. The condition is rare, but it is one which is likely to be met with now and then in coroners'-court cases; and if the coronary artery had given way more slowly it would have reproduced exactly those symptoms which we have already described in the account of a case of cardiac infarction followed by cardiac rupture.

